# Referee height influences decision making in British football leagues

**DOI:** 10.1186/s40359-020-0370-4

**Published:** 2020-01-17

**Authors:** Dane McCarrick, Gayle Brewer, Minna Lyons, Thomas V. Pollet, Nick Neave

**Affiliations:** 1 Department of Psychology, Faculty of Health & Life Sciences, Northumbria University, Newcastle upon Tyne, NE1 8ST UK; 2 School of Psychology, University of Liverpool, Liverpool, UK

**Keywords:** Height, Social dominance, Sport officials

## Abstract

**Background:**

Male height is positively associated with social dominance, and more agonistic/competitive behaviours. However, the ‘Napoleon complex’ or ‘small man syndrome’ suggests that smaller males are more assertive and punitive to compensate for lack of height and social dominance. Here, we assess possible relationships between height and punitive behaviours in a real-world setting.

**Methods:**

Using a non-experimental correlational design, we analysed data on 61 male association football referees from four professional leagues in England, and explored relationships between their height and punitive behaviours in the form of yellow cards, red cards and penalties given during an entire season.

**Results:**

Overall there was no effect of referee height on fouls awarded. However, there was a main effect of height on yellow cards awarded, with shorter referees issuing more yellow cards. The same effect was found for red cards and penalties, though this was moderated by league. In the lower leagues, more red cards and penalties were awarded by relatively shorter referees, but in the higher leagues more red cards and penalties were awarded by relatively taller referees.

**Conclusions:**

These findings from real-life public dominance encounters show that height is associated with punitive behaviours, but is sensitive to context.

## Background

Throughout the animal kingdom, body size has been associated with successful competition and the ability to obtain or maintain resources. Larger individuals are more likely to attain social dominance and thus enhance their ability to acquire resources and mates in the presence of others, either through agonistic or affiliate strategies [1, 2]. Consequently, body size has been coined an ‘honest’ biological marker for resource holding potential in non-human animals (for review, see [3]). In humans, body height is also associated with markers of physical quality such as strength or fighting ability [4, 5]. Taller men are also more likely to display aggressive behaviours [6]. Perhaps unsurprisingly then, taller men are perceived to be more dominant [7] and more leaderlike [8]. They also consider themselves to be more dominant than shorter rivals [9].

Height also influences a number of important outcomes. Taller men obtain higher starting salaries [10], higher overall income [11], and more promotions over the course of their career than do shorter men [12]. Consequently, taller men are more likely to hold positions of power, authority, and social status [13, 14], a trend that has been observed cross-culturally [2]. Most notably, taller presidential candidates receive more popular votes and are more likely to be re-elected than their shorter opponents [15]. Indeed, nonverbal cues that increase perceived status may do so by increasing the apparent size of the individual displaying them [16].

With regard to behavioural actions, it has been noted that men of greater stature are less sensitive to cues of dominance in other men [17]. Further, taller men respond with less jealousy towards socially and physically dominant rivals than their shorter counterparts [18] and (reflecting their physical strength) are more likely to win agonistic encounters [19, 20]. In a series of observational studies it was found that taller individuals were more likely to take precedence when entering a narrow passage only wide enough for a single person to fit through; were given more room on a narrow footpath than were smaller individuals; and were less likely to deviate from their walking gait when walking past a smaller male in a confined space [21]. In sporting contexts, taller players are perceived as committing more fouls than shorter players [22].

In contrast, it has been argued that shorter men compensate behaviourally for their height disadvantage, particularly when competing with same sex rivals. Increased aggression by shorter men has been termed the ‘Napoleon Complex’ or ‘Small Man Syndrome’ [23]. Smaller men may be particularly prone to aggressive behaviour if the desired reward exceeds the cost of losing the encounter, the cost of the display is relatively small, and assessment of resource holding power is not entirely accurate [24]. While the origins of this concept are unclear, the approach has been consistently linked to Adler’s inferiority complex theory, which assumes that people respond to feelings of inferiority on certain traits by overcompensating on others [25].

It has been revealed that shorter men show more indirect aggression in resource competitions with taller males, instead of direct or physical aggression [26]. When examining behavioural responses in illusory games described as tournaments to encourage competition, not only were smaller men more likely to keep more resources to themselves in a game in which they have all the power (dictator game) but did so within games whereby an opponent had some power (ultimatum game). This reinforces the view that shorter males exhibit greater behavioural flexibility in an attempt convey dominance they naturally lack in height. So, smaller men may employ more pronounced methods of incursion, potentially as a defence mechanism to give them the best chance to avoid the physical costs related to engaging in combat with a larger male. Such behaviour may be encouraged by the greater sensitivity to rivals displayed by shorter men [18, 27].

In order to assess the veracity of these two opposing viewpoints, it is important to focus on real-life behavioural situations for which punitive social decisions may have a powerful impact upon the individuals involved. One such setting in which it is possible to assess possible relationships between height and social dominance is that of a football (soccer) referee. Each match is controlled by a referee, who has full authority to enforce the ‘Laws of the Game’ by taking disciplinary action against players guilty of offences in connection with the match. Thus, it may be the case that well established masculine characteristics, such as physical height, may underpin a referee’s ability to both control games and deliver accurate decisions, given the abundance of literature that shows height to be a robust index of male dominance and authority [7, 13].

To our knowledge, there are few studies assessing height and refereeing decisions. One example, found that referees were significantly taller than assistant referees in a World Cup tournament, and in the French National League [28]. In the German League, taller referees were more likely to be appointed to prestigious games, perhaps reflecting the assumption that height is positively associated with competence. Interestingly, taller referees also awarded significantly fewer fouls with the authors concluding that “taller referees are better able to control the game by ‘bending their authority’, resulting in players committing fewer fouls”. These authors found no relationship between referee height and number of cards awarded however, but did not differentiate between yellow and red cards, or measure the number of penalties awarded [28]. Another study of the National Basketball Association found that relatively smaller referees called more personal fouls [29]. Together, these studies suggest that height is an important factor in refereeing decisions, with potential links to the “Napoleon syndrome”.

The aim of this study was thus to adopt the exploratory approach of relating referee height, with direct punitive decisions (in the form of fouls, yellow cards, red cards, and penalties) exhibited by the same referees in four professional English football leagues during the 2017/2018 season.

## Methods

### Design

The study adopted a non-experimental correlational design. The predictor variable was referee physical height measured in centimetres (cm). There were four response variables representing punitive behaviours exhibited by referees. These comprised the average number of fouls, yellow cards and red cards per game, as well as the total number of penalties awarded by each referee over the course of the 2017/2018 season in the English Premier League, Championship and Football League’s 1 & 2.

### Participants

61 male association football referees aged 26–53 years (*M* = 37.41, *SD* = 7.92) with an average height of 176.95 cm (*SD* = 9.81) volunteered to take part. Each was currently active within either the English Premier League, Championship or Football League’s 1 & 2, and officiated an average of 26.89 (*SD* = 6.19) games during the 2017/2018 season. The officials were classified in accordance with their referee level into three groups based on The Football Association National Referee Development Structure (see www.amateurfa.com/referees/promotion). Those operating within the Premier League (*N* = 18) were aged 33–53 years (*M* = 43.61, *SD* = 7.92), officiated an average of 21 games (*SD* = 5.74) during the season and had an average height of 178.06 cm (*SD* = 9.98). The Championship referees (*N* = 17) officiated an average of 28 games (*SD* = 3.41) during the season, were aged 28–48 years (*M* = 35.18, *SD* = 6.57) and had an average height of 175.94 cm (*SD* = 11.21). The League 1 & 2 referees (*N* = 26) officiated an average of 30 games (*SD* = 4.89) during the season, were aged 26–57 years (*M* = 34.57, *SD* = 7.42) and had an average height of 176.85 cm (*SD* = 9.03). Also, as referee age was significantly correlated with experience (measured via the total number of games they officiated during the 2017/18 season) (*r* = −.403, *p* < .001), age was also included in the analyses as a covariate.

### Materials

Data for each referee on fouls, yellow cards, red cards and penalties over the course of the 2017/2018 season was obtained from the online professional football analysis platform Wyscout (Wyscout.inc, 2018). A yellow card is given when a player acts with disregard to the danger to, or consequences for an opponent, and thus can be considered of intermediate punitive severity. A red card involves using excessive force against an opponent that exceeds the necessary use of force, resulting in the player being sent from the field of play. If a player receives two yellow cards in the same match they are automatically given a red card and ‘sent off’. Any of the above can result in the awarding of a penalty against a player if in their own penalty area. This involves the opportunity to take an unchallenged attempt at goal from 12 yards away, therefore resulting in the increased likelihood of scoring and therefore potentially winning the match.

As such, any decision made by a referee that significantly alters the likely outcome of a match is thus considered a ‘Key Match Decision’ by referee governing bodies [30]. These decisions predominantly consist of penalties and red cards and thus hold higher prestige as a punitive decision by referees. Across all leagues referees award significantly less penalties (*M* = 4.66, *SD* = 3.16) than they do fouls (*M* = 614.72, *SD* = 174.34), yellow cards (*M* = 85.23, *SD* = 25.51) and red cards (*M* = 4.23, *SD* = 2.96) (all *p* <. 001). Correspondingly, in contrast to fouls, yellow, and red cards that were examined in relation to the average number each referee awarded per game, to partial out the relative contribution of each game, penalty kicks were analysed in relation to the total number each referee had awarded during the season.

### Procedure

Following institutional ethical approval and written approval from the Football Association, data on fouls, yellow cards, red cards, penalties. Measures of height were obtained from the football analysis software Wyscout and UEFA (Uefa, 2018). When this information was not available on these platforms, and after giving their informed consent, referees provided their current physical height in centimetres via email or SMS message.

## Results

The data were analysed in R 3.5.1 via a series of Ordinary Least-Squares (OLS) regression models. The analyses are all provided in the Electronic Supplementary Materials (ESM) hosted on the Open Science Forum (https://osf.io/35b2j/) and contain full details and supplementary figures and analyses, such as Bayesian Regression Models [31].

### Descriptive statistics

Table 1 shows the descriptive statistics and correlations between the key variables.

**Table 1:** Means, standard deviations, and correlations with confidence intervals

Variable	*M*	*SD*	1	2	3	4	5	6
1. Height (cm)	176.95	9.81						
2. Age (years)	37.41	7.92	−.10 [−.34, .16]					
3. N Games	26.89	6.19	.04 [−.21, .29]	−.40** [−.59, −.17]				
4. Yellows per Game	3.16	0.61	−.28* [−.49, −.03]	−.14 [−.38, .11]	.11 [−.15, .35]			
5. Reds per Game	0.15	0.10	−.34** [−.54, −.09]	−.07 [−.32, .18]	.25 [−.00, .47]	.31* [.06, .52]		
6. Fouls per Game	22.74	3.26	−.11 [−.35, .15]	−.37** [−.57, −.13]	.17 [−.08, .41]	.44** [.21, .62]	.35** [.10, .55	
7. Penalties Awarded	4.66	3.16	−.16 [−.39, .10]	−.43** [−.62, −.20]	.44** [.21, .62]	.07 [−.18, .32]	.24 [−.01, .47]	.26* [.01, .48]

Note. M and SD are used to represent mean and standard deviation, respectively. Values in square brackets are the 95% confidence intervals for each correlation. * indicates *p* < .05. ** indicates *p* < .01

### Does height vary by division?

Table 2 shows the OLS regression models, which do not suggest that height varies significantly between divisions.

**Table 2:** Regression models for height

Model	Height (cm)	
	1	2
League: Championship	−.905 (3.102)	−.770 (3.093)
League: Premier	1.209 (3.050)	3.240 (3.481)
Age		−.225 (.188)
Constant	176.846^***^ (1.951)	184.617^***^ (6.786)
Observations	61	61
R^2^	.007	.031
Adjusted R^2^	−.027	−.020
Residual Std. Error	9.946 (df = 58)	9.909 (df = 57)
F Statistic	.200 (df = 2; 58)	.611 (df = 3; 57)

Notes

:^*^p < .05

^**^p < .01.

^***^ *p* < .001.

### Does referee stature relate to fouls given?

Height was not significantly associated with the number of fouls given (*F*_1,59_ = 0.694, *p* = .408, ESM). Age was negatively associated with the number of fouls given, with younger referees calling relatively more fouls (Adj. R^2^  .12, *F*_1,59_ = 3.055, *p* = .004, ESM).

### Does referee stature relate to the number of yellow cards per game

**Fig. 1:**
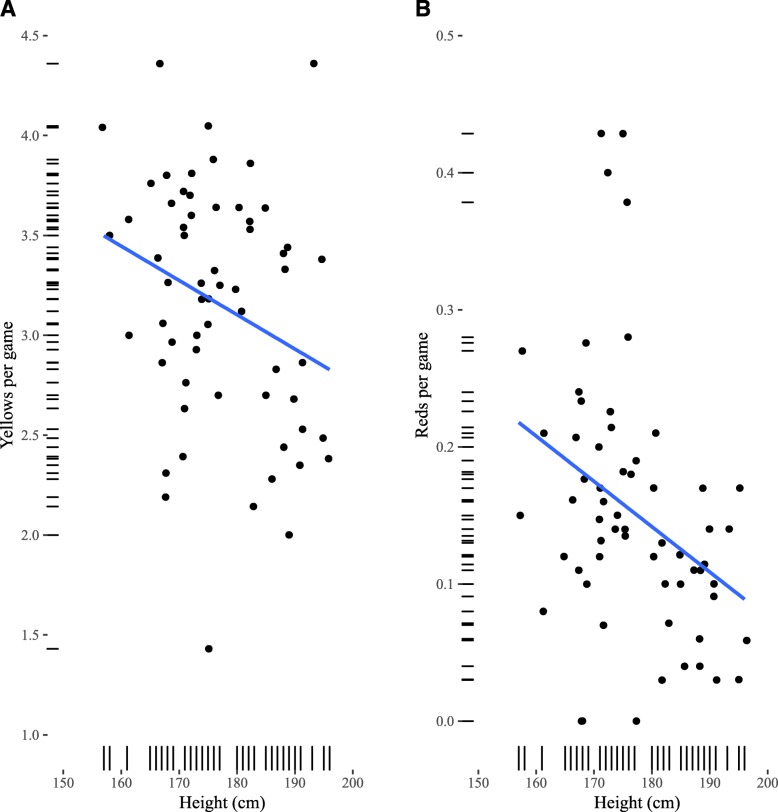
Number of cards per game (**a**: Yellow; **b**: Red) as a function of stature. Lines are OLS regression fits with 95% confidence intervals

**Fig. 2:**
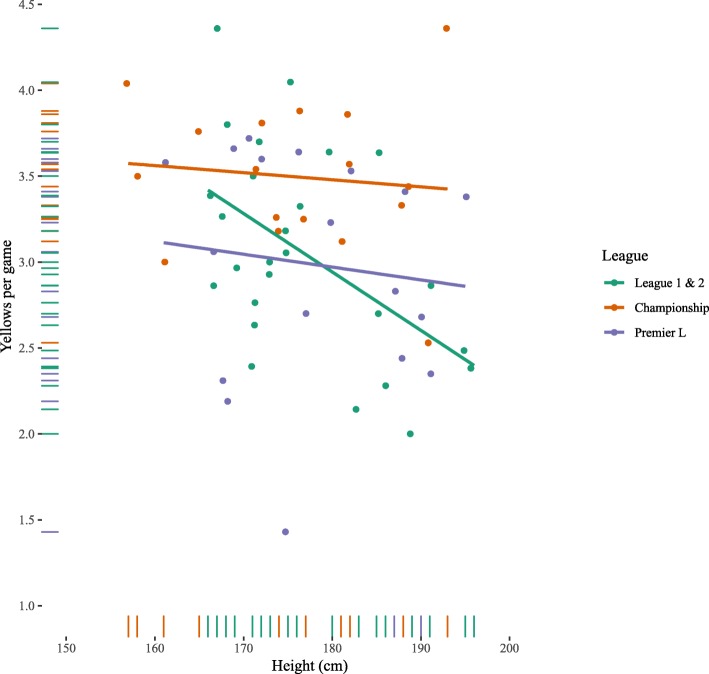
Number of yellow cards as function of height by league. Lines are OLS regression fits per league with 95% confidence intervals

Table 3 shows the OLS regression models for yellow cards. Height was negatively, and significantly, associated with the number of cards given (Fig. 1): shorter referees tended to give more yellow cards than taller referees. This relationship between height and yellow cards was upheld when accounting for age and division. The effect of height was also upheld when adjusting for number of fouls per game (B_height_ = −.014 (+/−.006), *p* = .048, Model in ESM). While there was no statistically significant interaction effect between league and height and the number of yellow cards per game (*F*_2,58_ = 1.74, *p* = .185; ESM), Fig. 2 suggests that this overall effect of height is due to the lower leagues (League 1 & 2).

**Table 3:** OLS Regression models for number of yellow cards per game

Model	Yellows per game			
	1	2	3	4
Height	−.017^*^ (.008)	−.018^*^ (.008)	−.016^*^ (.007)	−.034^**^ (.012)
Age		−.013 (.010)		
League: Championship			.432^*^ (.175)	−4.843 (3.077)
League: Premier			−.045 (.172)	−4.750 (3.237)
Height*League: Championship				.030 (.017)
Height*League: Premier League				.027 (.018)
Constant	6.193^***^ (1.372)	6.864^***^ (1.448)	5.832^***^ (1.314)	9.064^***^ (2.172)
Observations	61	61	61	61
R^2^	.077	.106	.190	.238
Adjusted R^2^	.061	.075	.147	.169
Residual Std. Error	.589 (df = 59)	.584 (df = 58)	.561 (df = 57)	.554 (df = 55)
F Statistic	4.918^*^ (df = 1; 59)	3.426^*^ (df = 2; 58)	4.449^**^ (df = 3; 57)	3.434^**^ (df = 5; 55)

Notes

^*^p < .05.

^**^ *p* < .01.

^***^ *p* < .001.

**Table 4:** OLS Regression models for number of red cards per game

Model	Reds per game				
	1	2	3	4	5
Height	−.003^**^ (.001)	−.003^*^ (.001)	−.003^**^ (.001)	−.003^**^ (.001)	−.007^***^(.002)
Yellows per Game		.036 (.020)			
Age			−.001 (.001)		
League: Championship				−.040 (.026)	−.803 (.427)
League: Premier				−.087^**^ (.026)	−1.791^***^ (.449)
Height*League: Championship					.004 (.002)
Height*League: Premier League					.010^***^ (.003)
Constant	.737^***^ (.213)	.512^*^ (.242)	.802^***^ (.227)	.742^***^ (.199)	1.510^***^ (.302)
Observations	61	61	61	61	61
R^2^	.114	.163	.125	.259	.413
Adjusted R^2^	.099	.134	.095	.220	.359
Residual Std. Error	.091 (df = 59)	.089 (df = 58)	.091 (df = 58)	.085 (df = 57)	.077 (df = 55)
F Statistic	7.593^**^ (df = 1; 59)	5.635^**^ (df = 2; 58)	4.139^*^ (df = 2; 58)	6.635^***^ (df = 3; 57)	7.733^***^ (df = 5; 55)

Notes

^*^p < .05

^**^p < .01

^***^p < .001

### Does referee stature relate to the number of red cards per game?

**Fig. 3:**
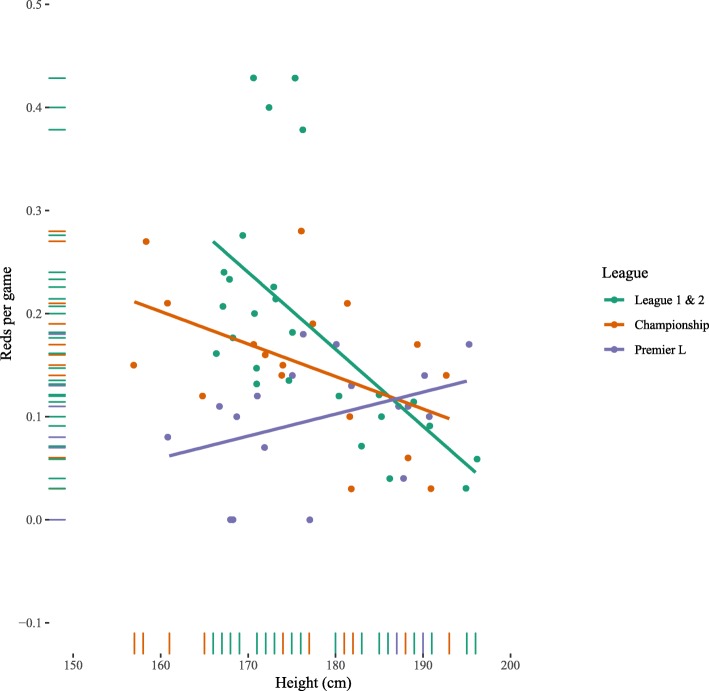
Number of red cards as function of height by league. Lines are OLS regression fits per league with 95% confidence intervals

Referee height was significantly and negatively associated with number of red cards per game Table 4 (Fig. 3). Given that we were unable to differentiate ‘straight’ red cards from red cards following a 2nd yellow card, we chose to include number of yellow cards as a control. This effect remained after controlling for number of yellow cards (Model 2), Age (Model 3) and Division (Model 4). The effect of height was also upheld when adjusting for number of fouls per game (B_height_ = −.003 (+/−.001), *p* = .009, Model in ESM). Interestingly, the model can be significantly improved by including the interaction effect between height and league (Model 5: *F*_2,55_ = 7.406, *p* = .001). Figure 3 shows this interaction effect, the negative association between height and number of cards is strongest in League 1 & 2, followed by the Championship. In the Premier League, the relationship is reversed, however, with a positive relationship between height and the number of red cards.

### Does referee stature relate to the number of penalties awarded?

**Fig. 4:**
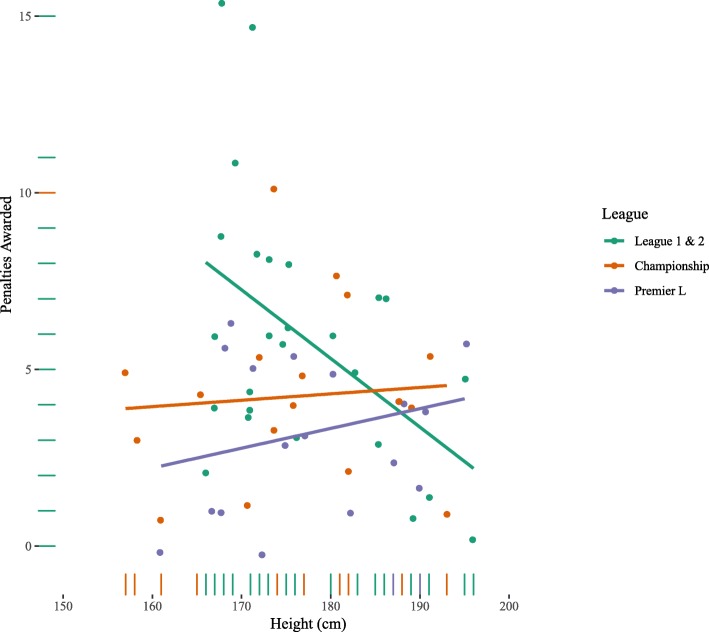
Number of penalties as function of height by league. Lines are OLS regression fits per league with 95% confidence intervals

Table 5 contains the OLS regression models. Model 1 shows that while height is negatively associated with penalties awarded, it is not statistically significantly so. However, there is evidence for an interaction effect between league and height (Models 4 and Model 5). Figure 4 illustrates this effect, in League 1 & 2 the relationship between height and penalties awarded is negative, whereas the relationship is positive for the Championship and Premiership. Thus, in the lower leagues, shorter referees tended to award more penalties, whereas in higher leagues (Championship/Premiership), taller referees tended to award more penalties. The ESM contains models which use a Poisson regression, treating the number of penalties awarded as a count variable, which leads to qualitatively similar conclusions as the OLS regression models.

**Table 5:** OLS Regression models for number of penalties

Model	Penalties awarded				
	1	2	3	4	5
Height	−.050 (.041)	−.064 (.037)	−.046 (.039)	−.194^**^ (.062)	−.180^**^ (.060)
Age		−.180^***^ (.046)			−.131^*^ (.053)
League: Championship			−1.730 (.930)	−39.234^*^ (15.627)	−31.899^*^ (15.257)
League: Premier			−2.645^**^ (.915)	−47.035^**^ (16.437)	−37.323^*^ (16.228)
Height*League: Championship				.212^*^ (.088)	.171 (.086)
Height*League: Premier League				.250^**^ (.092)	.202^*^ (.091)
Constant	13.540 (7.343)	22.759^**^ (7.019)	14.101^*^ (6.979)	40.289^***^ (11.027)	42.263^***^ (10.588)
Observations	61	61	61	61	61
R^2^	.024	.225	.157	.275	.347
Adjusted R^2^	.008	.198	.112	.209	.275
Residual Std. Error	3.149 (df = 59)	2.832 (df = 58)	2.979 (df = 57)	2.812 (df = 55)	2.693 (df = 54)
F Statistic	1.468 (df = 1; 59)	8.402^***^ (df = 2; 58)	3.532^*^ (df = 3; 57)	4.165^**^ (df = 5; 55)	4.788^***^ (df = 6; 54)

Notes

^*^p < .05

^**^p < .01

^***^p < .001

## Discussion

The present study examined the relationship between football referee height and punitive actions, in the form of fouls, yellow cards, red cards, and penalties awarded. Though there was no effect of referee height on fouls awarded, across leagues, shorter referees issued more yellow cards, indicating an inverse relationship between height and retributive decisions. For the harsher punitive gestures of red cards and penalties, context played an important role. In the lower leagues (League 1 and League 2), shorter referees issued more red cards and awarded more penalties than taller referees, consistent with their yellow card distribution. The opposite was true for the more prestigious Premier League and Championship, where taller referees issued more red cards and awarded more penalties than shorter referees. These findings demonstrate the importance of contextual influences, consistent with previous research investigating referee behaviour at different levels of competition [32].

It is possible that the more punitive behaviour by shorter referees in the lower leagues reflects greater incidence of punishable behaviour by the players (who may be less likely to view shorter referees as an authority figure). Indeed, researchers have proposed that taller football referees are subject to less challenging behaviour from the players, which would reduce their need to mete out punishments [28]. Another possible explanation is that because shorter males may lack the social dominance naturally afforded to taller males, they have to ‘stamp’ their authority and behave more punitively than would otherwise be expected [23, 24].

The more severe punitive decisions by taller referees in the higher leagues could be due to a combination between the high-status context, and other characteristics associated with aggression. In competition, less anxious [32], and more dominant men are more likely to aggress against their opponent after winning [33]. Height, as we know, is related to dominance [34], and also to the likelihood of refereeing in more prestigious games [28]. Although taller men may be overall less competitive with the members of their own sex [35], they may be more likely use sanctions as a form of competition in a high-stakes context. The Premier League and Championship do not only receive greater financial investment, but they also attract twice as many spectators than the lower leagues [36]. A potentially fruitful avenue for future studies would be to investigate the interactions between context (i.e., higher or lower stakes) and male height and dominance on aggression and intrasexual competition.

Of course, the greater rewards and scrutiny attached to the higher leagues may influence other factors aside from referee characteristics, such as the willingness of players or spectators to challenge decisions or derogate the referee. Indeed, the influence of crowd behaviour on referee decision-making is well-documented [37]. Anticipated player reactions may also influence willingness to penalise individuals. The risk of reprisals is a significant threat. In one study, all premier league referees surveyed reported being subjected to violent or abusive behaviour as a consequence of officiating [38]. The negative opinions of referees displayed by players and spectators [39] have persisted despite campaigns intended to promote respect [40, 41]. Though referees at all levels of competition may be subject to abuse, conflict experienced by referees at the higher levels is more likely to viewed by a large audience (e.g., larger crowds and televised), potentially leading to confrontation outside the match, wider ridicule, and a perceived loss of social status. Taller referees may feel more prepared to address such challenges or may be more motivated to protect their social status.

It should be noted that there are some limitations to our study. While our study is comprehensive in that it captures referees across four divisions, the absolute number of referees remains small, and unlike one study [28] we only examined one season. It should however, be borne in mind that a direct comparison between leagues, for example English Premier League vs. Bundesliga could be difficult. For example, our sample had on average 23 fouls per game, whereas the estimate for the Bundesliga was around 37 fouls per game [28]. Therefore, it is thus possible that the effect of height on refereeing behaviour varies substantially between international leagues. Another limitation relates to distinguishing between a red card given as a result of the accumulation of two lesser offences (two yellow cards) and a red card issued as a ‘straight red’, for a serious offence. The data we had access to did not differentiate between these ‘types’ of red cards and such information may prove to be interesting.

A further limitation relates to the fact that we were unable to ascertain the accuracy of the referee’s decisions, as the Wyscout platform does not provide this information. Future studies could compare referee decision-making accuracy as a function of height and league, using observational analysis of games and of course via the newly-employed Video Assistant Referee (VAR) system. Finally, our study has only focussed on one side of a dyadic interaction between referee and player, as we only measured referee behaviours and not player responses (these were not available in Wyscout). It could be the case for example that player response (e.g. verbal aggression) may vary according to their height differential between themselves and the referee, and this may itself vary between the different leagues. Future studies could perhaps assess this via observational analysis of player response during actual games.

## Conclusions

In conclusion, our study adds to the growing body of literature suggesting that male height is an important determinant of behaviour in competitive settings. Shorter referees issued more yellow cards and (in lower leagues) more red cards and penalties. Findings may reflect greater incidence of challenging behaviour from players or referee overcompensation for a lack of perceived dominance [23]. However, the influence of height on punitive actions is dependent on the context and in higher leagues taller referees issue more red cards and penalties. In higher stakes settings, taller men may be more likely to be motivated to maintain their position, which could have an influence on higher likelihood of influencing their decision-making and use of indirect aggression against others. The results of this study demonstrate how “the beautiful game” of football can be used in enhancing our understanding of male-male competition in real world situations.

## Data Availability

The dataset has been uploaded and raw data in anonymized form can be obtained from the corresponding author.
